# Impact of Carotid Artery Tortuosity on Technical Aspects of Endovascular Thrombectomy in a Newly Established Thrombectomy-Capable Stroke Center

**DOI:** 10.3390/clinpract15100183

**Published:** 2025-10-01

**Authors:** Katja Lovoković, Vjekoslav Kopačin, Mihael Mišir, Mateo Grigić, Domagoj Matijević, Tatjana Rotim, Domagoj Kretić, Damir Štimac, Anja Tomić, Lucija Čolaković, Tajana Turk

**Affiliations:** 1 Faculty of Medicine, Josip Juraj Strossmayer University of Osijek, 31000 Osijek, Croatia; klovokovic@gmail.com (K.L.); vkopacin@gmail.com (V.K.); mihaelmisir@gmail.com (M.M.); mateo.grigic@gmail.com (M.G.); domagojmatijevic@yahoo.com (D.M.); tatjana.rotim@gmail.com (T.R.); dkretic@gmail.com (D.K.); damir@stimac.org (D.Š.); anja.tomic93@gmail.com (A.T.); lucija.colakovic@gmail.com (L.Č.); 2 Department of Diagnostic and Interventional Radiology, University Hospital Centre Osijek, 31000 Osijek, Croatia; 3 Department of Neurology, University Hospital Centre Osijek, 31000 Osijek, Croatia; 4 Poliklinika Medikol, 31000 Osijek, Croatia; 5 Department of Radiology, Dr. Juraj Njavro National Memorial Hospital, 32000 Vukovar, Croatia

**Keywords:** carotid artery, internal, ischemic stroke, thrombectomy

## Abstract

**Background/Objectives**: Blood vessel tortuosity can complicate endovascular procedures such as endovascular thrombectomy in acute ischemic stroke. This study aimed to assess the morphometric characteristics of carotid arteries and investigate the association between the tortuosity of the carotid arteries and the technical aspects of endovascular thrombectomy, patient demographics and clinical characteristics, and treatment outcome. **Methods**: This retrospective study included 84 patients with ischemic stroke treated by endovascular thrombectomy at the newly established thrombectomy-capable stroke center. The following data were collected from prethrombectomy computed tomography angiography: aortic arch type, type of carotid artery tortuosity, and tortuosity index (TI). The technical aspects of the procedure, as well as patient demographics, were collected from the radiological information system. **Results**: Time from arterial puncture to the first pass was significantly shorter in patients with a nontortuous carotid artery compared to a tortuous one (*p* = 0.006). There were no significant differences in the number of passes, total duration of the procedure, and the difference in National Institutes of Health Stroke Scale (NIHSS) score before and after the procedure regarding the form of tortuosity. Patients with hypertension had significantly higher tortuosity index values compared to those without hypertension (*p* = 0.008), and patients with a nontortuous carotid tree were significantly younger compared to those with all forms of tortuosity (*p* = 0.003). **Conclusions**: The majority of patients had tortuous carotid arteries, which were associated with older age and hypertension. A high index of tortuosity was associated with a longer time from arterial puncture to the first pass, but not to the treatment outcome. Preprocedural recognition of carotid artery tortuosity may aid in endovascular thrombectomy procedural planning.

## 1. Introduction

Ischemic stroke, which can be caused by arterial thrombosis, embolic event, or arterial dissection, with subsequent decrease in tissue perfusion, is the second leading cause of death and disability worldwide [1]. Ischemic stroke is effectively treated with reperfusion therapy, which consists of intravenous thrombolysis and endovascular thrombectomy [2]. Early reperfusion therapy can rescue the neuronal tissue within the ischemic penumbra by achieving timely reperfusion of viable tissue, allowing patients to have better functional outcomes [3]. During endovascular thrombectomy procedures, different endovascular devices are used to gain access to the occlusion site, where the thrombus can be extracted and the vessel recanalized [4]. However, successful recanalization is not always possible. Unfavorable vascular anatomy and arterial tortuosity, which are common in elderly patients with acute stroke, can prolong the procedure time and reduce the probability of successful recanalization of the blood vessel [5]. Arterial tortuosity is a change in the morphology and anatomy of blood vessels in the form of wall thickening, reduced elasticity, lumen reduction, flow changes, kinking, and coiling. It is associated with aging, hypertension, hyperlipidemia, diabetes, atherosclerosis, and genetic and environmental factors. A patient’s arterial tortuosity can make endovascular material positioning more difficult and time-consuming, which prolongs the procedure and can negatively influence the treatment outcome [6]. Evaluation of the technical outcome of the procedure is performed immediately after the procedure, using the mTICI score (Modified Thrombolysis In Cerebral Infarction) which describes the extent of recanalization and reperfusion of the occluded vessel [4]. When managing patients with acute stroke, the National Institutes of Health Stroke Scale (NIHSS) is the most commonly used scale to assess stroke severity and neurological deficit. The NIHSS allows for a rapid neurological assessment, can predict outcomes, and can help set realistic treatment goals. Also, it can be used for evaluating immediate treatment outcomes by comparing NIHSS scores before and after treatment. The scale scores individual impairments, which are then summed and range from 0 to 42, where higher scores indicate a more severe stroke [7]. The objective of this study was to analyze the morphometric characteristics of carotid arteries In patients with acute ischemic stroke and to examine the relationship between the tortuosity of the carotid arteries with the technical aspects of endovascular thrombectomy, as well as with patient demographics, clinical characteristics, and treatment outcome.

## 2. Materials and Methods

### 2.1. Patients

A retrospective review of all consecutive patients who underwent endovascular thrombectomy for acute ischemic stroke at the Department of Diagnostic and Interventional Radiology at the University Hospital Centre Osijek, Croatia, from November 2020 until February 2024 was performed. Ethical approval for this study was obtained from the Institutional Review Board of University Hospital Centre Osijek, Croatia, (Approval No. R1-4642/2024, 23 April 2024) and from the Faculty of Medicine Osijek Ethics Committee of Josip Juraj Strossmayer University od Osijek (Approval number 2158-61-46-24-17, approved on 5 February 2024). Only patients who had transfemoral endovascular thrombectomy for M1 occlusion were included in this study. The exclusion criteria were bilateral transfemoral or transbrachial approach, tandem occlusion, intracranial angioplasty or stenting, and incomplete or inadequate imaging studies. Among 217 consecutive patients, 84 patients met the criteria to be included in the study. An institutional Radiological Information System (RIS) was used to collect the following data: baseline demographics, comorbidities, occlusion location, puncture-to-first pass time, total procedure time, number of passes, mTICI score, and NIHSS score before and 24 h after thrombectomy.

### 2.2. Arterial Tortuosity Measurement and Definition

Prethrombectomy computed tomography angiography (CTA) images were evaluated using dedicated software (Sectra IDS7 version 22.2, Sectra AB, Linkoping, Sweden). Specially trained medical students (K.L., L.Č.) performed the analysis and measurements under the supervision of two radiologists (T.R., D.K.) with more than 10 years of experience. All of the assessors were blinded to the outcomes of the patients’ treatment.

The following data were collected: aortic arch type, type of carotid artery tortuosity, and tortuosity index (TI).

Aortic arch types were analyzed and defined according to well-known classification by Shuford et al. [8] as follows: type 1 (distance between the horizontal line at the origin of brachiocephalic trunk and horizontal line at the apex of aortic arch is < diameter of CCA); type 2 (distance between the horizontal line at the origin of brachiocephalic trunk and horizontal line at the apex of aortic arch is 1–2 times diameter of CCA); and type 3 (distance between the horizontal line at the origin of brachiocephalic trunk and horizontal line at the apex of aortic arch is >2 times diameter of CCA). The presence of bovine arch (common origin of brachiocephalic trunk and left CCA) was also noted.

**Figure 1:**
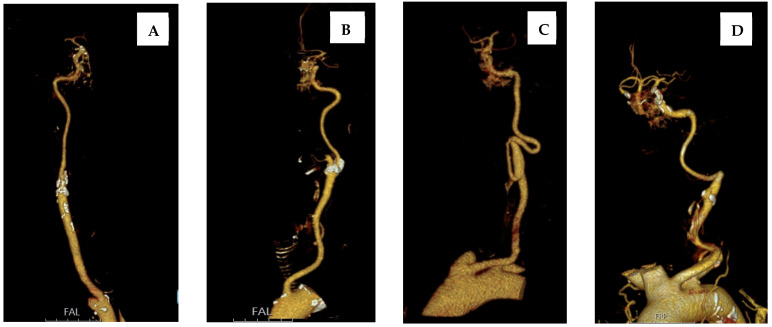
Types of extracranial carotid artery tortuosity: (**A**) straight; (**B**) tortuous; (**C**) coiled; and (**D**) kinked.

Types of extracranial carotid artery tortuosity were assessed—as previously described by Nagata at al.—as straight, tortuous, coiled, and kinked [9], as seen in Figure 1.

Tortuosity index was defined by previously established formula [4] as follows (Equation (1)):

Tortuosity index (TI) = ((actual length/straight length) − 1) × 100(1)

**Figure 2:**
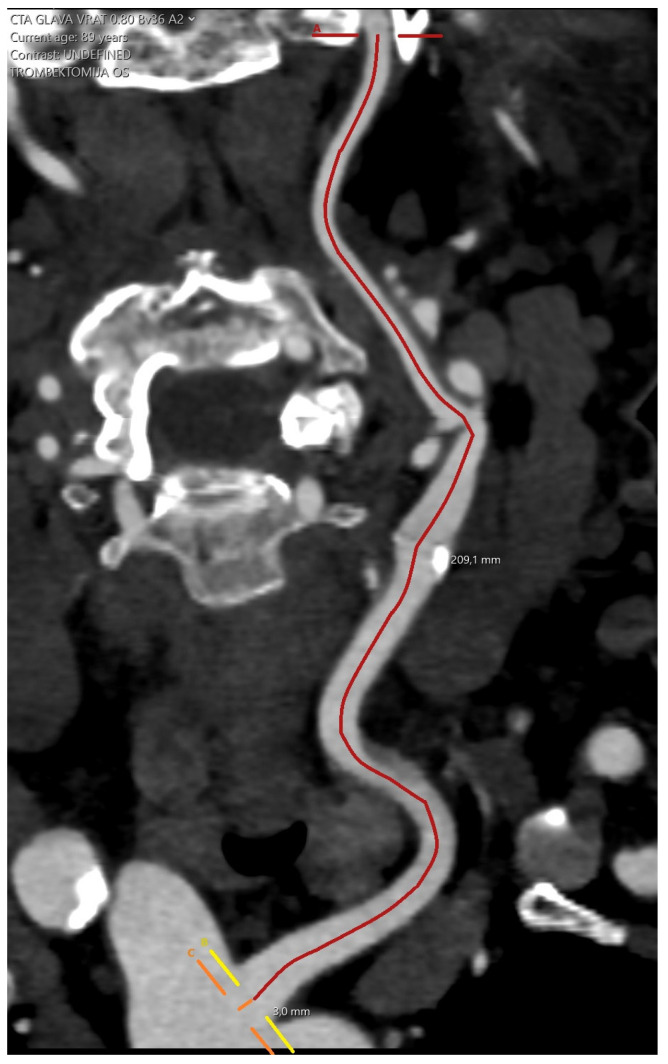
Measuring the actual arterial length.

The actual arterial length was defined as the length between the origin of the ipsilateral artery at the aortic arch and the skull base, measured using Vessel Analysis application of the Sectra software, where centerline points were placed along the artery at 1 cm increments (Figure 2).

**Figure 3:**
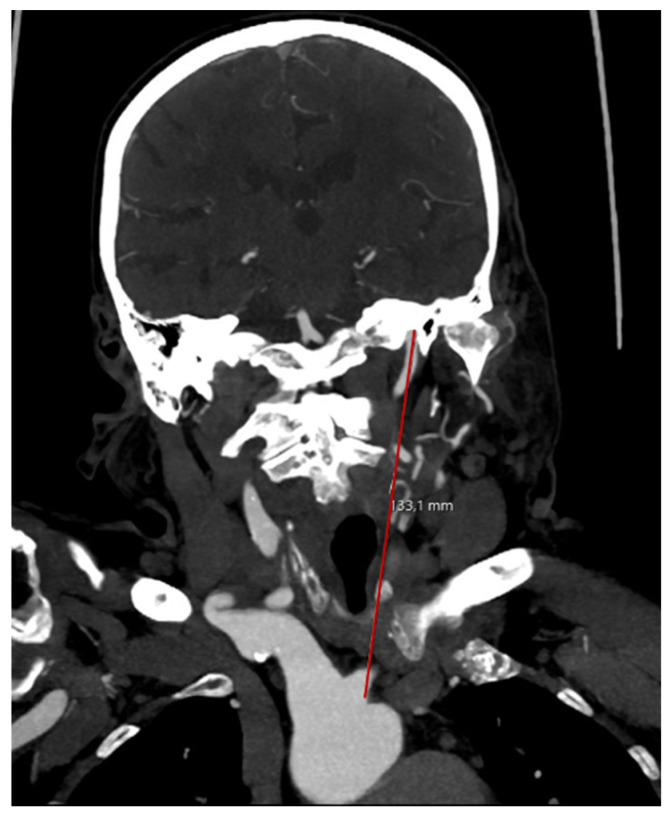
Measuring the straight arterial length.

The straight length was defined as the direct measurement between origin of the ipsilateral artery at the aortic arch and skull base on a coronal image reconstruction (Figure 3).

### 2.3. Statistical Analysis

Categorical data were presented as absolute and relative frequencies. Differences in categorical variables were tested using the Chi-square test. The normality of the distribution of numerical variables was assessed using the Shapiro–Wilk test. Numerical data were described using the median and interquartile range (IQR). Differences in numerical variables between two independent groups were analyzed using the Mann–Whitney U test (with the Hodges–Lehmann median difference and 95% confidence interval of the difference reported), while differences among multiple independent groups were assessed using the Kruskal–Wallis test (with post hoc Conover analysis). The association between continuous variables was evaluated using Spearman’s rank correlation coefficient ρ (rho). All analyses assumed no differences across carotid tortuosity groups, with statistical tests applied to assess potential variations in demographics, clinical factors, procedures, and outcomes. In cases of multiple pairwise comparisons, *p*-values were adjusted using the Bonferroni correction to control for inflation of type I error. All *p*-values were two-tailed. The significance level was set at alpha (α) = 0.05. Statistical analyses were performed using MedCalc^®^ Statistical Software version 22.018 (MedCalc Software Ltd., Ostend, Belgium; https://www.medcalc.org (accesses on 15 May 2024)).

## 3. Results

### 3.1. Patient Characteristics

A total of 84 patients (31 men [36.9%] and 53 women [63.1%]), median age 76 years (interquartile range 67–83 years), met the inclusion criteria for this study. The most common comorbidities were arterial hypertension (81%), atrial fibrillation (52%), and hyperlipidemia (43%). Forty-four patients (52.4%) presented with left-sided M1 occlusion and forty patients (47.6%) with right side M1 occlusion (Table 1).

### 3.2. Technical Aspects of Endovascular Thrombectomy

The median number of passes was two (interquartile range 1–3) and the median time from arterial puncture to the first pass was 25 (interquartile range 16.3–33). Total procedure time was between 6 min and 165 min (median 47 min). The median NIHSS at admission was 15 (interquartile range 12–17), whereas the median NIHSS 24 h post thrombectomy was 9 (6–14) (Table 2).

It was observed that older patients had higher tortuosity index values (Rho = 0.351), as higher values are also present in patients with a longer time from puncture to the first pass (Rho = 0.291). There was no significant association of tortuosity index with the total duration of the procedure, or the values of the NIHSS (Table 3).

Patients with a type 1 aortic arch were significantly younger, with a median age of 61 years (interquartile range from 58 to 75 years), compared to all other types of aortic arch (Kruskal–Wallis test, *p* = 0.01). Meanwhile, there are no significant differences in the number of passes, the time from puncture to the first pass attempt, the total duration of the procedure, and the difference in NIHSS score before and after the procedure with regard to the shape of the aortic arch (Table 4).

Patients with straight carotid trees were significantly younger, with a median age of 63 years (interquartile range from 46 to 73 years), compared to all other forms of tortuosity. Patients with a tortuous carotid artery were also significantly younger than patients with carotid artery kinking (Kruskal–Wallis test, *p* = 0.003). The time from puncture to the first pass attempt was significantly shorter in subjects with a straight carotid tree compared to all other forms of tortuosity (Kruskal–Wallis test, *p* = 0.006). There are no significant differences in the number of passes, the total duration of the procedure, and the difference in the NIHSS score before and after the procedure with regard to the form of tortuosity (Table 5).

Although the values of the tortuosity index are slightly higher at the higher values of the mTICI scale, the difference, although it exists, is not significant (Table 6).

Patients with arterial hypertension have significantly higher values of the tortuosity index compared to those without arterial hypertension (25.33 vs. 15.29) (Mann–Whitney U test, *p* = 0.008), while there are no significant differences in other comorbidities or with regard to smoking difference (Table 7).

## 4. Discussion

Ischemic stroke is highly prevalent and is among the main causes of death and disability worldwide. Since it is effectively treated with endovascular thrombectomy, it is important to investigate the association of changes in carotid tree anatomy with the technical aspects and clinical outcomes of the procedure, as well as with the demographics and clinical characteristics of patients.

This study was conducted on 84 patients who underwent endovascular thrombectomy for the treatment of acute stroke, more specifically, patients with the occlusion of the M1 segment of the middle cerebral artery, which is the most common site of occlusion in ischemic stroke [8]. The study was conducted in a newly established thrombectomy-capable stroke center, and the procedures were performed by specially trained interventional radiologists with previous experience in non-neurointerventional procedures.

Of the 84 patients in this study, there were more women than men. The influence of gender on the incidence of stroke varies by age group; in younger patients, the incidence is similar in both sexes, in patients between 45 and 74 years of age, the incidence is higher in men, and in patients older than 74 years, women have a higher incidence of stroke. This can be explained by the fact that women have a longer life expectancy and thus a higher probability of suffering from a stroke. Also, due to their older age when a stroke occurs, they generally have more comorbidities than men. Men, on the other hand, consume alcohol and tobacco products more often, which is a risk factor for the development of atherosclerosis and consequent stroke [9].

Considering the location of the occlusion, in our study, the occlusion of the M1 segment of the left, rather than the right, middle cerebral artery was somewhat more common. According to the available medical literature, ischemic strokes caused by the occlusion of blood vessels of the left hemisphere are more common and have a more severe clinical presentation, worse functional outcome, and higher mortality [10]. Research has shown that there are differences in hemodynamics between the left and right carotid arteries, which is attributed to a higher flow velocity in the left carotid artery, consequently causing greater wall shear stress, outward arterial remodeling, and damage to the intima, which can result in atherosclerosis and possibly more severe ischemic events [11]. Also, the left common carotid artery is a direct branch of the aortic arch, and emboli have an easier path to the distal branches [10].

In this study, a significantly shorter time from the arterial puncture to the first pass was recorded in patients with a straight carotid tree. The association of a higher tortuosity index with a longer time from puncture to the first pass was also shown, but no significant association of the tortuosity index with the total duration of the procedure and the number of first pass attempts was demonstrated. The explanation for this difference is that the influence of the tortuosity of the arterial tree is greatest during the initial placement of a long introducer or guiding catheter in the internal carotid artery, and after the aforementioned devices are placed, the tortuosity of the carotid tree no longer has such an influence. By prolonging the duration of the procedure, in the case of multiple attempts to extract the thrombus, the proportion of time required for the initial placement of the material through the tortuous arteries is reduced. On the other hand, in the research conducted by Koge et al., patients with pronounced tortuosity required a greater number of passes and a longer total duration of the procedure [12]. This difference in results may be due to the fact that the mentioned group of authors included in their research patients with other occlusion sites, while our research is limited only to patients with occlusion of the M1 segment of the middle cerebral artery, which is also technically the simplest endovascular thrombectomy procedure. Research conducted by Kaymaz et al. showed that there is a strong association between the tortuously altered anatomy of the supraaortic arteries and a longer time to access the internal carotid artery, and a longer time to access the internal carotid artery is associated with a lower rate of successful recanalization [13]. Although our research also showed that with increased tortuosity, the success of recanalization according to the mTICI scale is decreased, the difference was not statistically significant. From a technical point of view, it takes more time to reach the occlusion site through tortuous arteries because the maneuverability of the material changes when passing through multiple turns of tortuous arteries, especially if relatively rigid materials are used, although newer materials can more adequately follow the arterial anatomy.

This research showed the expected correlation of the index of tortuosity with older age of the subjects. Age-associated degenerative changes and reduced elasticity of the vessels, combined with mechanical injury to the arterial wall, which is caused by the forces of blood flow that vessels are constantly exposed to, play an important role in vessel tortuosity development [14].

Aging and reduced elasticity are also important factors in elongation of the thoracic aorta and the aortic arch. Aortic arch elongation can be classified in three types, third being the most elongated [15]. In our study, subjects who had a type 1 aortic arch were significantly younger than patients with all other forms of aortic arch, as well as subjects who had a straight or slightly tortuous carotid tree compared to kinking blood vessels. Those results are in line with our expectations, because we do not expect significant elongation of the aortic arch or tortuosity of the arteries in younger patients.

The NIHSS scores were used to assess the clinical outcome of the procedure. NIHSS scores were calculated before and 24 h after the procedure. It is expected for NIHSS values to lower after reperfusion, meaning that the initial deficit decreased. At admission, NIHSS scores ranged from 7 to 27, and 24 h after the procedure ranged from 0 to 40 points. There was no significant correlation between tortuosity index, tortuosity type, aortic arch type, and NIHSS values in our study. In the research conducted by Kurmann et al. NIHSS scores at admission ranged from 9 to 19, and after 24 h from 3 to 15 points. Patients with better reperfusion results had lower NIHSS scores after 24 h [16]. In our study, mTICI scores were used to assess the technical aspect of the procedure, scoring reperfusion, with 0 being no reperfusion and 3 being complete reperfusion. In the aforementioned study by Kurmann et al., all patients with an mTICI score of 0 had NIHSS scores over 16 points, and almost half of the patients who achieved reperfusion mTICI score 3 had 0 to 3 points on the NIHSS. They proved that measurement of the NIHSS score after 24 h has the strongest predictive value for early and long-term survival and that low NIHSS scores after 24 h reduce the probability of long-term mortality [16]. In the research conducted by Dargazanli et al. patients who achieved near-complete or complete mTICI 2c/3 reperfusion were shown to have better functional outcomes compared to patients who achieved partial mTICI 2b reperfusion [17]. Hassan et al. proved that achieving mTICI 2c/3 reperfusion in the first attempt at thrombus extraction (first pass effect) is significantly associated with better clinical and functional outcomes and with reduced patient mortality [18]. In our study, satisfactory reperfusion was not achieved in 11% of patients (mTICI 0/1). Reasons for failure of reperfusion vary from difficulty in attempting to access cervical or intracranial blood vessels to the inability to dislodge and extract the thrombus even though the site of occlusion has been successfully accessed. Other explanations may be various comorbidities of blood vessels, such as vasculitis or intracranial atherosclerosis, or thrombi of different composition, such as calcified or neoplastic thrombi [19].

In the research conducted by Koge et al., the rate of achieving a mTICI score of 2c/3 was significantly lower in patients with a tortuous carotid tree compared to those with a straight one [12]. In our research, although the values of the tortuosity index are slightly higher with the higher values of the mTICI scale, the difference, although it exists, is not significant.

This study examined clinical and demographic characteristics that put patients at greater risk for stroke. Among the patients, the most prevalent characteristic associated with a higher risk was arterial hypertension, while only a small number of patients smoked. The low percentage of smokers can be attributed to the fact that the data in this study were taken retrospectively, and in the urgency of admitting such patients, no emphasis was placed on their lifestyle, i.e., there were probably more smokers than what was recorded in the medical documentation. Subjects with arterial hypertension had significantly higher tortuosity index values compared to those who did not have arterial hypertension, while there were no significant differences in other comorbidities or with regard to smoking. Research conducted by Huang et al. showed that age and duration of hypertension are two risk factors for the development of carotid artery tortuosity and that the use of antihypertensive drugs can reduce the incidence of carotid artery tortuosity in hypertensive patients [20].

The limitations of this study are the relatively small sample of patients and the fact that the data were collected retrospectively. Further research on larger patient samples is needed to accurately determine the impact of carotid tree tortuosity on the technical aspects and clinical outcomes of endovascular thrombectomy, as well as the impact of comorbidities and patient lifestyles on the development of vessel tortuosity. Another limitation of this study is the reliance on bivariate analyses, which cannot fully adjust for potential confounding factors. Future larger-scale studies should apply multivariable methods to confirm whether the associations observed here are independent.

## 5. Conclusions

This study has shown that the majority of patients suffering from stroke who were eligible for endovascular thrombectomy had tortuous carotid arteries, which were associated with older age and hypertension. A high index of tortuosity was associated with a longer time from arterial puncture to the first attempt at thrombus extraction, but not to the treatment outcome. Preprocedural recognition of carotid artery tortuosity may aid in endovascular thrombectomy procedural planning.

## Figures and Tables

**Table 1:** Baseline patient demographics.

Characteristics	Number (%) of Patients
Sex	
Male	31 (36.9)
Female	53 (63.1)
Atrial fibrillation	44 (52)
Arterial hypertension	68 (81)
Diabetes mellitus	17 (20)
History of ischemic stroke	15 (18)
Hyperlipidemia	36 (43)
History of myocardial infarction	10 (12)
History of TIA ^1^	7 (8)
Current smoker	7 (8)
Active COVID-19 ^2^ infection	7 (8)
History of COVID-19 ^2^ infection	7 (8)
Lesion location	
Right-sided M1 ^3^ occlusion	40 (47.6)
Left-sided M1 ^3^ occlusion	44 (52.4)

^1^ TIA—Transient ischemic attack; ^2^ COVID-19—Coronavirus disease 2019; ^3^ M1—M1 segment of middle cerebral artery.

**Table 2:** Mean and dispersion measures of subjects’ age, number of attempts, time to first pass, total procedure time, and NIHSS scores.

	Median (Interquartile Range)	Minimum– Maximum
Patient age	76 (67–83)	37–93
Number of passes	2 (1–3)	0–7
Time from arterial puncture to the first pass (minutes)	25 (16.3–33)	5–107
Total procedure time (minutes)	47 (27–66)	6–165
NIHSS ^1^ at admission	15 (12–17)	7–27
NIHSS ^1^ 24 h after EVT ^2^	9 (6–14)	0–40
Difference in NIHSS ^1^	−5 (−10–−0.25)	−18–23

^1^ NIHSS—National Institutes of Health Stroke Scale; ^2^ EVT—endovascular thrombectomy.

**Table 3:** Correlation of tortuosity index with age, number of passes, procedure duration, and NIHSS score (Spearman’s rank correlation coefficient).

	Spearman’s Rank Correlation Coefficient Rho Tortuosity Index (*p*-Values)
Patient age	0.351 (0.001)
Number of passes	−0.097 (0.38)
Time from arterial puncture to the first pass (minutes)	0.291 (0.01)
Total procedure time (minutes)	−0.058 (0.61)
NIHSS ^1^ on admission	0.141 (0.20)
NIHSS ^1^ 24 h after EVT ^2^	−0.037 (0.74)
Difference in NIHSS ^1^	−0.170 (0.12)

^1^ NIHSS—National Institutes of Health Stroke Scale; ^2^ EVT—endovascular thrombectomy.

**Table 4:** Correlation between age, number of passes, time from puncture to the first pass, and total procedure duration, as well as the difference in NIHSS score in relation to aortic arch type.

	Median (Interquartile Range) Considering Aortic Arch Type				*p* *
	Type 1	Type 2	Type 3	Arcus Bovinum	
Age	61 (58–75)	77 (68–83)	82 (74–83)	75 (69–84)	0.01 ^†^
Number of passes	2 (1–3)	2 (1–3)	1 (1–2)	2 (1–3)	0.50
Time from arterial puncture to the first pass (minutes)	25 (17–32)	23 (15–29)	26 (16–45)	28 (21–33)	0.51
Total procedure time (minutes)	42 (25–50)	48 (24–74)	40 (25–57)	59 (42–80)	0.20
Difference in NIHSS ^1^	−5 (−10–−2)	−7 (−10–−3)	−5 (−8–0)	−3 (−10–3)	0.34

* Kruskal–Wallis test (post hoc Conover). ^1^ NIHSS—National Institutes of Health Stroke Scale. ^†^ at the level of *p* < 0.05 there are significant differences: Type 1 vs. (Type 2, Type 3, Arcus bovinum).

**Table 5:** Correlation between age, number of passes, time from puncture to the first pass and total duration of the procedure, as well as the difference in NIHSS score values in relation to the type of tortuosity.

	Median (Interquartile Range) Considering Tortuosity Type				*p* *
	Straight	Tortuous	Coiling	Kinking	
Age	63 (46–73)	75 (63–82)	77 (68–84)	81 (74–85)	0.003 ^†^
Number of passes	2 (1–3)	1 (1–2)	2 (1–3)	1 (1–3)	0.91
Time from arterial puncture to the first pass	16 (12–17)	28 (20–34)	26 (22–31)	25 (15–39)	0.006 ^‡^
Total procedure time	45 (19–70)	49 (27–66)	50 (31–73)	48 (32–70)	0.82
Difference in NIHSS ^1^	−5 (−10–−0.5)	−5 (−10–−3)	−3 (−9–0)	−5 (−12–0)	0.70

* Kruskal–Wallis test (post hoc Conover). ^1^ NIHSS—National Institutes of Health Stroke Scale. ^†^ at the level of *p* < 0.05 there are significant differences: Straight vs. (all the rest); Tortuous vs. kinking. ^‡^ at the level of *p* < 0.05 there are significant differences: Straight vs. (all the rest).

**Table 6:** Correlation of mTICI scale values with tortuosity index.

mTICI ^†^ Score	Median (Interquartile Range)	*p* *
	Tortuosity Index	
0	18.18 (8.43–29.34)	0.72
1	16.47 (5.65–22.25)	
2a	13.11 (−3.79–33.65)	
2b	20.1 (8.14–31.11)	
2c	10.75 (6.45–30.73)	
3	21.37 (12.54–31.7)	

* Kruskal–Wallis test. ^†^ Modified Thrombolysis in Cerebral Infarction.

**Table 7:** Differences in the tortuosity index considering comorbidities and smoking.

	Median (Interquartile Range) of Tortuosity Index Considering Characteristics		Difference	95% Confidence Interval	*p* *
	No	Yes			
Atrial fibrillation	15.29 (9.22–22.45)	25.33 (11.48–32.89)	2.89	−0.11 to 12.01	0.06
Arterial hypertension	11.74 (4.52–19.25)	20.40 (10.98–31.20)	7.76	2.09 to 14.93	0.008
Diabetes	18.18 (9.24–30.01)	22.50 (16.11–32.16)	4.94	−2.23 to 11.26	0.18
History of stroke	18.18 (9.25–29.56)	23.78 (11.34–31.99)	3.2	−4.19 to 10.27	0.38
Hyperlipidemia	18.95 (9.64–31.57)	18.66 (9.68–26.63)	−0.29	−6.26 to 5.35	0.92
History of myocardial infarction	18.77 (9.43–30.73)	16.61 (10.75–25.52)	−0.59	−8.95 to 6.72	0.89
History of TIA/CAS ^1^	18.18 (9.25–29.56)	25.70 (15.11–36.41)	6.59	−4.20 to 16.9	0.17
Smoking	19.21 (10.04–30.83)	10.75 (4.30–23.49)	−7.41	−18.01 to 3.51	0.18

* Mann–Whitney U test. ^1^ TIA/CAS—transitory ischemic attack/carotid artery stenting.

## Data Availability

The data presented in this study are available on request from the corresponding author due to specifics of the patients.

## Abbreviations

The following abbreviations are used in this manuscript:

TI	Tortuosity index
NIHSS	National Institutes of Health Stroke Scale
mTICI	Modified Thrombolysis In Cerebral Infarction
RIS	Radiological Information System
CTA	Computed tomography angiography
CCA	Common carotid artery
IQR	Interquartile range
TUA	Transient ischemic attack
COVID-19	Coronavirus disease 2019
EVT	Endovascular thrombectomy
CAS	Carotid artery stenting
